# Involuntary psychiatric treatment during the COVID-19 pandemic. An international qualitative study

**DOI:** 10.3389/fpsyt.2023.1200888

**Published:** 2023-05-25

**Authors:** Agostino Carbone, Martin Knapp

**Affiliations:** CPEC - Care Policy and Evaluation Center, Department of Health Policy, London School of Economics and Political Science, London, United Kingdom

**Keywords:** mental health care, involuntary admission, psychological distress, psychological interveniton, COVID-19

## Abstract

**Background:**

During the COVID-19 pandemic, studies report that in the first few months of the lockdown there was a decrease in requests for mandatory psychiatric treatment, while, in contrast, following the second wave, the number of cases increased. This study investigates the use of compulsory psychiatric treatments internationally in the first and subsequent phases of the pandemic.

**Methods:**

Sixteen key people were interviewed: eight mental health care professionals and eight scholars in Italy, Greece, China and Chile. Participants were asked to discuss their experience of the motivations, diagnoses and management of patients undergoing an involuntary psychiatric hospitalization.

**Results:**

The analysis through Grounded Theory highlighted four themes: (a) the culture of psychiatric care services, (b) the effect of the pandemic on involuntary hospitalizations, (c) exceptional management of hospitalization, and (d) policies and suggestions for more inclusive mental health treatments.

**Conclusion:**

During the first wave, respondents reported a decrease in the use of involuntary treatments, while a gradual increase was seen in the following months. Italy extended compulsory psychiatric treatment to a group of new users, including young people and adolescents with acute crises; in other contexts, the main users are chronic psychiatric patients.

## 1. Introduction

The COVID-19 pandemic has seriously affected the daily lives of the global population, heavily compromising the physical and psychological well-being of the whole world and placing a strong burden on Public Health Care (1–3). Recent psychological and psychoanalytic studies on the emotional effects of COVID-19 (4–8) show that living with the pandemic has led to tiredness and uncertainty, derived from not being able to count on the usual certainties based on an order that guarantees the predictable and certain functioning of a series of organizational and contextual components. In addition, the loss of confidence in the health system and a loss of credibility concerning the regulations issued by the institutions has generated a serious sense of anomie. The various pandemic waves have undoubtedly caused a pervasive feeling of anxiety about being infected, and the drastic reduction in socialization experiences due to virus containment measures have certainly increased the risk factors for mental distress in the general population, as well as in people from the most fragile sections of society, such as those with pre-existing psychiatric diagnoses or those suffering from relational and behavioral disorders (3, 5, 9–11).

Recent scientific papers show that psychiatric and emotional disorders were significant risk factors during the pandemic in terms of physical and mental health (5, 11); people suffering from psychiatric disorders seem to be at high risk of Infection due to pre-existing disorders, unhealthy lifestyle, cognitive deficiency or reduced level of consciousness of the risk (5, 10, 12). Data on access to mental health services suggest a significant increase in mental health consultations (including telephone consultations and e-consultations) (13, 14), an increase (15) certainly attributable to a rise in the number of cases of acute crisis in old and new users as a consequence of the experiences of anguish and isolation resulting from the pandemic (9, 16), and of the weakening of community mental health care networks.

Regarding the use of mandatory psychiatric treatments during the first year of COVID-19 pandemic, early studies (17, 18) indicate a reduction in the number of compulsory psychiatric treatments during the first wave of the pandemic, unlike in the second, when they increased again. A slight decrease of treatments are not clearly attributable to a reduction in acute crises, notwithstanding contextual variables including a greater tolerance on the part of family members who preferred not to report acute cases to avoid referrals to hospital structures for fear of exposing themselves to the risk of infection from the health care or paramedic staff, and the temporary closure of psychiatric hospital wards in certain countries (especially in Latin America) most affected by the pandemic, or unprepared to face it. Data also indicate an increase in the average length of hospital stay during the period from March to June 2020 compared to the previous year, probably due to the difficulty in guaranteeing a “safe” return home for the patients (17, 19) a condition that in some cases violates the principles of brevity and transience that characterize compulsory treatments. The studies carried out so far on the progress of compulsory psychiatric treatments during the pandemic (17–26) have been the first appreciable attempt by scholars and mental health professionals to monitor and make sense of what they witnessed in terms of the mental health of the population while the pandemic was ongoing and gradually advancing around the world.

### 1.1. The current research

The aim of this study is to investigate the topic of mental health interventions following the onset of emergency situations and acute crises that led to hospitalizations during the COVID-19 pandemic. The objective of this research is to collect information on the use and trend of mandatory psychiatric treatments during the first two waves of the COVID-29 pandemic in different areas of the world that differ in the degree of progress of territorial psychiatric reform through (1) the direct experiences of professionals (psychiatrists and clinical psychologists) and (b) the studies and considerations of key eminent local scholars in the fields of clinical psychology, psychiatry and mental health policy.

The specify aim are: (a) to understand whether and how the pandemic has increased the practice of involuntary treatments, and whether the emergency produced by the spread of COVID-19, (b) to value the capacity (in term of resource and culture of psychiatric intervention) of the different health systems to manage and process a user when was requested an immediate intervention, (c) explore any extraordinary measures put in place in psychiatric wards or hospitals for the treatment and rehabilitation of patients subject to involuntary treatment, and (d) to consider the ethics of mandatory psychiatric treatments (25) during the pandemic globally.

## 2. Materials and methods

### 2.1. Participants

The participants were sixteen (*n = 16*) key people in the field of compulsory psychiatric hospitalizations during the pandemic. Specifically, eight (8) university professors and eight (8) mental health professionals were interviewed. Participants were selected from people the researcher’s knew and through a snowball sampling strategy. The interviewees were specifically selected from four different countries – Italy, Greece, Chile and China (Hong Kong) – in order to gain an understanding of their experiences regarding a global phenomenon such as the pandemic. In Italy, (a) two psychiatrists director of a department of mental health in the Friuli Venezia Giulia area were interviewed, and (b) a psychiatrist-psychoanalyst director of a mental health unit in a district of Rome, (c) a psychiatrist working in Psychiatric Unit “SPDC” of a public Hospital in Naples and (d) a professor of clinical psychology in Center Italy were interviewed. In Greece, (e) one university professors of psychiatry, (f) a professor of clinical psychology, (g) a psychiatrist from a psychiatric hospital in Thessaloniki and (h) a psychiatrist from a psychiatric hospital in Athens were interviewed. In Chile, (i) two university professors of Clinical Psychology and (l) two psychiatrists from a psychiatric hospital in Santiago de Chile were interviewed. In China, (m) three university professors of Mental Health Policy in the district of Hong Kong were interviewed. There were six women and ten men. The sampling was completed when the theoretical saturation of the categories that emerged during the interviews in relation to the research question was reached.

### 2.2. Instrument for data gathering

The data was collected through an area-focused narrative interview. The choice to gather data according to interviews allow to capture people making sense of their social experience and of their own role in it (27–29). In last decades several method has been developed to realize narrative data analysis according both to bottom up strategies (30–33) and top down strategies (34). Our approach was informed to grounded theory (35, 36) specifically we developed an area-focused narrative interview built in both a top-down (theoretical) manner, starting from emerging topics in the literature, and a bottom-up method starting from the researcher’s experience in the field. The interview explored the following topics: (a) the functioning and use of mandatory psychiatric treatment in their country, (b) the use of mandatory psychiatric treatments during the pandemic, including changes in the number of admissions and causes of mental illness, and the management of patients and vulnerable groups, and (c) proposals to reduce involuntary psychiatric admissions. The questions allowed participants to express their own point of view and the associations connected with what was requested as much as possible. The researcher’s interventions were limited to encouraging the participants to continue talking in moments of silence and embarrassment. In Chile and Greece, the interviews took place in pairs (a psychiatrist together with a university teacher) to deal with linguistic and cultural mediation problems. The interviews were carried out in English and Italian.

### 2.3. Procedures

The interviews were carried out between July and August 2021; the participants were contacted by email and telephone, and appointments were made in locations that were convenient for them. The interview that took place in Rome was carried out in-person at one of the workplaces of the interviewees. The other interviews were conducted online. The average duration of the interviews was approximately 90 min. The interviews were conducted by an expert interviewer with previous experience in the field of qualitative research. After establishing contact with the participants, the researcher obtained informed consent from them after explaining the objective of the research, how the data would be shared, and how anonymity would be ensured. The entire procedure was approved by the university ethics committee.

### 2.4. Data analysis

The collected interviews were transcribed verbatim, and subsequently analysed using the Grounded Theory Method (37, 38) with the help of Atlas.ti software (39) for the analysis of textual data. The analysis process took place in three phases through bottom-up interpretative models in the direction of an ever-greater abstraction (40). The analysis was conducted by a researcher with direct experience and training in the area of qualitative research in mental health and clinical psychology. The data were coded and categorized through a constant comparison of the research questions with the gradual emergence of meanings through the data. The hypotheses regarding the relationship between the codes, the potential categories and any sources of bias have been included in the comments. During the entire process of reading and searching for meaning in the data, a reflective method and a bottom-up approach were used that directed attention to the subjective experiences within which it is embodied, giving meaning to the participants’ experience (40). In the first phase (open coding), 25 codes were assigned; in the second phase (axial coding), the number of codes was reduced (to 10 codes) through a criterion of similarity of meaning in eleven categories. Finally, in the third phase (selective coding) 4 categories were further grouped through an inductive process into more abstract macro-categories of meaning (themes).

## 3. Results

The results of the qualitative study are condensed into four key themes. These are: (a) *the culture of psychiatric care services*, (b) *the effect of the pandemic on involuntary admissions*, (c) *exceptional management of hospitalization in order to reduce the spread of infection, and* (d) *policies for more inclusive mental health care.* The themes are presented and argued below. For each theme, a table with the most relevant sentences (quotations) extracted from the interviews with the key characters is has been added to support the discussion and the interpretation of results.

### 3.1. (a) The culture of psychiatric care services – first theme

The first theme immediately highlights the characteristics that distinguish the different cultural models of intervention in the confrontation of mental illness. In recounting how the healthcare system deals with cases of acute crisis during the pandemic, the interviewees highlighted and highlighted the models within which involuntary treatments are implemented and the conception of the person affected by mental illness. Territorial reforms of national psychiatric systems are in various stages in different countries. In Chile, as in Greece, the reform has not been completed, there are mixed systems, there are still asylums converted into modern psychiatric hospitals even if they use an intervention model that is no longer purely of the asylum type, there is still a stigma towards person who manifests mental distress and the lack of a mental health prevention perspective. In Greece, the number of involuntary psychiatric treatments exceeds the number of patients treated voluntarily, and it has the highest rate of compulsory psychiatric treatments in Western Europe (41–45). In Italy, on the other hand, a reform of the psychiatric system, starting in the 1970s, has largely been implemented thanks to the Basaglia reform (46, 47). The situation in Hong Kong is very different from the others; although it is now part of China, the psychiatric model used is the one adopted in the UK There are many differences between this city and the rest of China (see Table 1).

**Table 1:** First Core Category *“The culture of psychiatric care services*” with relative descriptive quotations from participants.

(a) The culture of psychiatric care services	“In Thessaloniki there are 8 psychiatric units: 5 belonging to psychiatric hospitals, 3 are psychiatric units in general hospitals and 3 are in universities. Usually, involuntary patients will end up going to psychiatric hospitals, while voluntary patients will go to clinics” (Professor of Clinical Psychology Thessaloniki, GR).
	“In Chile we have a mixed form of care, there are still 4 psychiatric hospitals and some psychiatry wards in general hospitals. In the last year, a reform is accelerating the closure of the former asylums” (Psychiatrist, CL).
	“Data show that around 73% of people evaluated in emergency clinics come voluntarily. Among them, 67% are evaluated and only 29% hospitalized. Among the unvoluntary patients: around 88% are hospitalized while 9.5% are only evaluated. There is a big difference among clinics as some hospitals tend to hospitalize everyone” (Professor of Clinical Psychology Thessaloniki, GR).
	“Here in Friuli Venezia Giulia, the place where Basaglia operated, we have a community-based intervention model, so it is very difficult to carry out mandatory psychiatric treatment, there are home teams that go and reside at the psychiatric patient’s home until the urgency is over” (Psychiatrist, Friuli Venezia Giulia, IT).
	“In Rome there is a psychiatric model that over the years has developed and nourished itself from a system that was organized starting from the Basaglia reform that established a form of community assistance with mental discomfort. Furthermore, the presence of psychoanalysis within the working groups of professionals made us think that behind every involuntary hospitalization there is a family problem. For us, our users are not just hospitalized people. In our centers there are psychological bonds between family members, psychiatrists and hospitalized people, this allows us to treat family discomfort” (Psychiatrist, Rome, IT).

### 3.2. (b) The effect of the pandemic on involuntary hospitalizations – second theme

The second theme collects information about access to mandatory psychiatric treatments in the countries under consideration. The trends proposed here are the result of the experience of the professionals in service during the period explored and of the preliminary studies carried out by the scholars at the universities of the community to which they belong.

All respondents agree that in the first phase of the pandemic, the number of involuntary patients decreased, and then increased in the following months globally. In countries such as Chile and Greece and China, the people who underwent forced hospitalization were people suffering from serious problems and chronic psychiatric disorders, while in Italy also people suffering from depression or anxiety crises. In all contexts analyzed, it emerges that people subjected to compulsory psychiatric treatment were often people who lived alone and could not count on assistance from family members, further increasing the sense of feelings of despair and loneliness. In addition, Italian mental health professionals also report that even young people who have had a first acute crisis during the pandemic have resorted to compulsory psychiatric treatment, and therefore not only chronic patients.

Another factor that the interviewees identified as having contributed to the onset of emergency situations was the suspension of all prevention and territorial assistance activities carried out in person by the mental health worker, this abruptly interrupted home care and inhibited all consultative activities such as psychotherapy, psychological counseling, drug monitoring, etc.

It should also be noted that in some situations, such as in Chile and China, the mental health centers were closed for a few weeks, while in Italy and Greece, after the first days of disorientation, face-to-face activities were replaced by of e-mental health care (see Table 2).

**Table 2:** Second core category “*The effect of the pandemic on involuntary* admission*”* with more descriptive quotations from participants.

(b) The effect of the pandemic on involuntary admission	“During the pandemic, we noticed a difference between the first four months of the pandemic and then the following months: March–June 2020: There were very strict rules in Greece and people were complying with regulations. During this time, admissions rate dropped to nearly 0%, including involuntary admission. After Summer 2020: Once people started getting used to the pandemic, numbers started rising again and we went back to our number admissions rates (60% involuntary admissions)” (Psychiatrist, Thessaloniki, GR).
	“We did not notice any connection between the Covid-19 crisis and delirious people. However, it is probably too soon to see the repercussions that the pandemic must have had people’s mental health. In any case, it is likely that the current situation will trigger different disorders like anxiety and depression” (Psychiatrist, Athens GR).
	“Another problem is about the shame associated with mental illness and often people or their family members prefer patients to be admitted far from their house” (Professor of Clinical Psychology Thessaloniki, GR)
	People who have undergone compulsory psychiatric treatment are often people who live alone and cannot rely on family resources.
	“According to a study conducted before the pandemic, we noticed a difference between patients admitted voluntarily and those involuntarily. The first ones are usually married or living with someone or in a community; while patients admitted against their will are usually living alone, not married, are usually men and show aggressive or psychotic behavior. Age does not seem to be an interesting factor” (Professor of Clinical Psychology Thessaloniki, GR).
	“We had very strict rules and no visitors were allowed. Unfortunately, as we did not have public Wi-Fi, only people with their own devices could easily contact their relatives. Also, some psychiatric patients are not allowed to keep their phones so that was harder for them to contact their relatives. To cope with this situation, we increased instances in which patients could have access to their phones and talk to their relatives” (Psychiatrist, Thessaloniki, GR).

### 3.3. (c) Exceptional management of hospitalization in order to reduce the spread of infection – third theme

*Concerning the organization of the patients’ hospitalization within the psychiatric ward, the risk of contagion, the interviewees report, has required a major reorganization of the accesses and reception of psychiatric patients. Everyone agrees on the fact that the period of hospitalization has usually been longer than normal, this because on the one hand the families of origin were reluctant to welcome into their home a patient who had been hospitalized in a hospital, where the risk of contagion was certainly greater, on the other hand, testing the patient during the access and hospitalization period made the treatment path longer. Furthermore, in other cases it has happened that patients tested positive at the time of admission or have contracted the COVID-19 virus in the ward, making the period of hospitalization much longer. In addition, in order to minimize the risk of contagion in the hospital new specific role and strategies for a safer management of patients were establish. First, it was necessary to undergo a molecular test upon admission and to live in isolation for a few days before the response, the management was complicated for same people with severe diagnosis who did not understand the need to be isolated awaiting test results. For infected people, as in Italy, special areas were set up within the hospital psychiatry departments (within general hospitals), in other situations, however, patients were placed in general hospitals, because psychiatric hospitals did not guarantee adequate treatment for lung infection caused by the virus. On the other hand, in China, positive patients have been placed in “other” structures, other than psychiatric or general hospitals, thus increasing their experience of isolation and alienation* (see Table 3).

**Table 3:** Third core category *“exceptional management of hospitalization in order to reduce the spread of infection”* with more descriptive quotations from participants.

(c) Exceptional management of hospitalization in order to reduce the spread of infection	“We worried that schizophrenic patients would not cope well with Covid-19 regulations, especially with Covid-19 tests. However, patients seemed to cooperate well, even psychotic people who usually are paranoic and do not like to be touched” (Psychiatrist, Athens, GR).
	“During the second wave of the pandemic, we had two rooms (one for female and one for male) in which incoming patients would be put in quarantine until their Covid-19 results were available. In case of positive test results, those patients with no symptoms would be moved to another department, while those with symptoms would be sent to Covid-19 hospitals” (Psychiatrist, Thessaloniki, GR).

### 3.4. (d) Policies and suggestions for more inclusive mental health care – forth theme

The future of health policies for mental health, according to the interviewees, features some priorities across the different cultures of existing mental health services. In the first instance, one of the interviewees suggested the construction of mental health centers in different areas of their country, particularly in rural areas and islands, as a strategy to improve patient care. This was in Greece, where reform around the locations of mental health centers is still incomplete. According to the Greek expert, decreasing the concentration of psychiatric patients and involuntary psychiatric treatments in some hospitals in large cities such as Athens and Thessaloniki would allow better patient management and less crowding of urban psychiatric hospitals (45). A second and long-standing problem that respondents highlighted is the underfunding and scarcity of human resources within the mental health area. In particular, one of the interviewees indicated that one improvement would be to reduce the gap between public and private and allow even the poorest segments of the population to access the same treatments. An Italian interviewee hopes for greater funding from the state, which currently spends only a small portion of its budget on mental health. Another important point was the need to create a future for people with mental illness. In particular, there is a need to create a real strategy to reintegrate those with mental illnesses, especially young people, back into society. In many countries, there are no strategies for entering the world of work, and no policies are planned for the reintegration of people suffering from mental illness. The mentally ill are often considered unable to work, but this is a concept that should be changed, and the policy should provide preferential routes to work placement after hospitalization in the mental wards. This suggests a focus is needed on the training of psychiatric, nursing and psychological staff who work within mental health centers. If there are realities where positive models of intervention have been tested (48), these should be disseminated beyond the local reality (see Table 4).

**Table 4:** Fourth core category “*Policies and suggestions for more inclusive mental health care”* with more descriptive quotations from participants.

(d) Policies and suggestions for more inclusive mental health care	A big problem is that our hospital accepts patients from half of Greece as some regional hospitals still do not accept involuntary patients. Larger mental health centers should also be opened in rural areas of Greece and on the numerous islands of Greece.” (Psychiatrist, Thessaloniki, GR).
	“The health system should hire new staff, here in Italy the National Health System spends only 3% of its budget on mental health, they must hire qualified personnel, psychiatrists and psychologists ready to use a non-medical treatment model and based on rehabilitation intervention and psychotherapy” (Psychiatrist, Friuli Venezia Giulia, IT).
	“The big problem is the gap between the public and private systems, here in Greece many professionals are conniving with the current system, we need to fight, create alarmism and give the poor a chance. Here the marginal bands are abandoned to themselves, more funding is certainly needed”(Professor of Psychiatry, Athens, GR).
	*“The biggest problem is giving people suffering from psychiatric distress a future. The question of finding a job for them is crucial to their development. Whenever I can, I try to convince the shopkeepers to hire some of my former users, especially if they are young, but you understand, this must be a goal of the state, not left to the goodwill of psychiatrists”(Psychiatrist, Rome, IT).*
	*“It would be useful for a psychiatric model of community to spread throughout China, it will be very difficult, but we hope that the new generations of professionals will be able to spread a humanized psychiatric culture, the role of universities will be fundamental for this purpose (Professor of Mental Health, Hong Kong).*

## 4. Discussion and conclusions

The research has highlighted some key aspects of the use of mandatory psychiatric treatments during the COVID-19 pandemic. The interviewees provided some details on the health policies in force in the areas they belong to and, in other cases, information on the field about their direct experience in the psychiatric services of the national health system. This has made it possible to trace the boundaries and the socio-cultural framework within which psychiatric intervention and the treatment of patients in critical situations have been conceived and implemented in an unprecedented anomic condition, such as the spread of the COVID-19 Pandemic.

First of all, we saw a decrease in involuntary treatments during the first months of the pandemic; this is transversal data confirmed by all the interviewees. This phenomenon should not be read superficially. It is possible that during a period of hard lockdown, families were more tolerant of the mental illness of one of their family members, for fear of getting infected by coming into contact with health personnel or while gaining access to hospitals. In fact, the fear of coming into contact with the health system led to a reduction in psychiatric hospitalizations in general in the months of March and April, and consequently also involuntary hospitalizations. The decision not to hospitalize people who had previously suffered from mental illness was a choice of both families and health personnel to reduce the risk of contagion among the population. It is also probable that disadvantaged people could still count on their own resources, not yet drained by the exhausting prolongation of the pandemic. In the following months, starting from summer 2020, the trend seemed to return to the levels prior to the pandemic. In Greece, we are told a slight increase in involuntary hospitalizations has been registered when compared to those of 2019. This is most likely due to an easing of safety measures and less fear of contagion.

Another interesting topic concerns the causes of hospitalization. Here, differences emerge between the countries and the local realities to which the interviewees belong. In Greece and Chile, for example, our interviewees reported that the people who were involuntarily hospitalized were those suffering from severe psychiatric disorders, such as schizophrenia and paranoid and bipolar personality disorders. There was no difference between before and after the pandemic, as only people suffering from severe mental illness, very often already known to family members or local health personnel, are involuntarily admitted. In these countries, the emergence of new psychopathologies and other causes during the pandemic is not highlighted. The consideration that derives from this is that, in this conception, mental distress coincides with a severe psychopathology that affects the body and mind of a person, and the person is identified with his or her own psychopathology. On the other hand, the two psychiatrists interviewed in Italy speak of the use of compulsory involuntary treatments not in relation to a severe psychopathology of a person, but of critical events in general which then triggered the use of hospitalization. These events include the discomfort of living all together with the family during the lockdown which, for people who were not used to sharing close family relationships in confined spaces, during the pandemic resulted in attacks of violence against themselves and members of the family or community. This difference between Italy, Greece and Chile speaks to us of the differences in the conception of mental illness between the different countries. In Chile and Greece, the psychiatric reform has not yet been completed, so a conception of mental distress as a psychopathology persists; a concept of mental distress as a failure of the relationship between contextual dimensions, social relations and the individual is still developing. Italy, on the other hand, has been the protagonist since the 1970s of a very profound reform of both the national psychiatric system, which has led to the complete closure of asylums, and the concept of psychiatric discomfort, which has moved from an organicistic dimension to a dimension of psycho-socio-relational. In Italy, it was possible to identify a new discomfort that emerged during the pandemic, a discomfort that seems to have affected the youth segment of the population, who saw themselves deprived of their future, their relationships with peers, and their chance to attend school or university. A conflict has emerged between youth culture and family culture, experienced by the young people in an obligatory and asphyxiated way during this period. Another issue concerns the management of patients who are involuntarily hospitalized in psychiatric structures. In Italy, there were no psychiatric wards for people affected by COVID-19. Rather, in each ward, distinct spaces of isolation were organized for the first days of hospitalization to find out the outcome of COVID tests. In other contexts, such as in Chile and Greece, some specific areas of psychiatric hospitals were used as COVID-19 wards, where infected people spent part of their hospital stay if positive. In these last two cases, the segregation of psychiatric patients who were separated within the psychiatric structure was certainly greater. On the other hand, in all the situations analysed, there was a lack of staff to manage the admission and hospitalization of patients.

At the end of the interviews, the participants were asked to indicate the objectives that, in their opinion, the psychiatric services of their country should pursue to improve the management of mental distress and reduce the number of people having mandatory psychiatric treatments. It was interesting to note that each reality was expressed differently, but their wishes were similar: to increase the number of psychiatric structures, to increase the number of professional staff within the psychiatric system, and to increase financing of mental health.

Another central point that can guide the development of the psychiatric systems analyzed concerns the models through which mental distress is read and interpreted. Without a model that understands the emergence of mental suffering within relationships, we are destined to make mental illness chronic. Reform of the psychiatric system should concern the possibility of intervening in specific contexts and reducing the risk for vulnerable groups of the population, such as immigrants, the poor, and young people. In conclusion, we would like to make a final reflection on the subject of compulsory psychiatric treatments. One wonders whether intervening in these cases necessarily corresponds to a forced hospitalization in a psychiatric facility or if it is possible to carry out other types of treatment, for example by carrying out a home intervention over a medium-short period (days or a few weeks). Forced hospitalization is a violent tool for those who are subjected to it, and often the patient’s rehabilitation measures do not allow them to find a home with their family that can accommodate them once hospitalization is over (49–51) The hope for the future is to be to be able to nourish and nurture a global model of community mental health intervention (52) by training professional staff in a holistic conception of mental distress and social stigma (53–55), also educating society to request and integrate psychiatric and psychosocial interventions (56) with support from the third sector, ensuring the continuity of care in the family and community after discharge from hospitals.

## 5. Limitation

This research certainly has important limitations, of which the reader should be aware. The first limit certainly concerns the small number of participants, it was not easy to find participants during the sampling period, many universities were closed, many professionals were difficult to find internationally via email, although efforts in this direction have been made. The second limitation concerns the difficulty of finding professional personnel in the field of mental health in China, despite our great interest, we have not received further interest from mental health professionals to join this study in that moment; in fact it was difficult to finds personal information of professionals through the web due to language barriers. Unfortunately, local scientific literature is still scarce (57–59), there are no studies or data on mandatory psychiatric treatments in China or that collect experiences of mental health professionals. Another limitation concerns the fact that this exploratory study does not specifically concern mental health in childhood or adolescence (56). Another limitation concerns the generalizability of the data, the results of this study are to be understood exclusively in relation to a specific non-replicable historical moment and that the experiences of the key people involved are to be considered as subjective narratives not attributable in general to universal contexts. Finally, another limit concerns the choice of countries to compare, a choice dictated by availability within the acquaintance of the authors and the desire to compare different continents (Europe, Asia and South America), with great divergence on the treatment of mental illness and in the advancement of a territorial reform of mental health treatment; this choice could certainly have concerned other National contexts.

## Data availability statement

The raw data supporting the conclusions of this article will be made available by the authors, without undue reservation.

## Ethics statement

The studies involving human participants were reviewed and approved by the Ethics Committee of LSE - London School of Economics and Political Sciences (Ref. 28888 - 2021). The patients/participants provided their written informed consent to participate in this study.

## Author contributions

AC and MK contributed to conception and design of the study. AC organized the database, performed the analysis, and wrote the first draft of the manuscript. MK supervised the manuscript. All authors contributed to the article and approved the submitted version.

## Funding

This work was founded by CPEC at Department of Health Policy of London School of Economics and Political Sciences.

## Conflict of interest

The authors declare that the research was conducted in the absence of any commercial or financial relationships that could be construed as a potential conflict of interest.

## Publisher’s note

All claims expressed in this article are solely those of the authors and do not necessarily represent those of their affiliated organizations, or those of the publisher, the editors and the reviewers. Any product that may be evaluated in this article, or claim that may be made by its manufacturer, is not guaranteed or endorsed by the publisher.
